# Development and evaluation of a subjective measurement of geographical hardness-to-reach: a survey of data collectors in a large-scale cluster randomised trial of azithromycin mass drug administration

**DOI:** 10.7189/jogh.16.04322

**Published:** 2026-11-06

**Authors:** Juho Luoma, Fadima Cheick Haidara, Oumar Samake, Laura Adubra, Mahamadou Abdou, Dagmar Alber, Ulla Ashorn, Elaine Cloutman-Green, Mamadou Diallo, Jane Juma, Camilla Ducker, Yue-Mei Fan, Gwydion Gruffudd, Lotta Hallamaa, Rikhard Ihamuotila, Owen Martell, Collins Okello, Tiia Haapaniemi, Taru Vehmasto, Akshaya Murugesan, Cheick Amadou Tidiane Traore, Kaisa Ylikruuvi, Nigel Klein, Per Ashorn, Samba Sow, Yin Bun Cheung

**Affiliations:** 1 Center for Child, Adolescent, and Maternal Health Research, Faculty of Medicine and Health Technology, Tampere University and Tampere University Hospital, Tampere, Finland; 2 Center for Vaccine Development–Mali, Bamako, Mali; 3 Ministère de la Santé et du Développement Social du Mali, Bamako, Mali; 4 Great Ormond Street Institute of Child Health, University College London, UK; 5 Great Ormond Street Hospital, London, UK; 6 Tro Da Ltd, Caerphilly, Wales, UK

**Keywords:** hard-to-reach, accessibility, survey, health equity

## Abstract

**Background:**

Distance from residence to the nearest health facility is a known predictor of child mortality. However, physical distance alone may not fully capture geographical hardness-to-reach. Subjective assessments from data collectors may offer additional insight into village-level accessibility and its relationship to health outcomes.

**Methods:**

This was a sub-study of Large-scale Assessment of the Key health-promoting Activities of two New Mass Drug Administration regimens with Azithromycin, a large-scale cluster-randomised trial of mass administration of azithromycin in Mali. We developed a 14-item survey and administered it to 25 data collectors to evaluate village geographical accessibility. We recorded survey completion time and respondent burden assessments to investigate the measurement’s feasibility. We assessed convergent validity by correlation between two composite scores derived from survey items and objective measures (distance and travel time to the nearest health facility, the Malian national outreach programme status, and the average monthly rainfall) and predictive validity using under-five mortality as the criterion. Inter-rater reliability was evaluated by intra-cluster correlation coefficients (ICC).

**Results:**

The mean time of completing the survey was three minutes. Survey composite scores reflecting subjective village inaccessibility were associated with higher under-five mortality in the villages: one standard deviation change towards harder access associated with up to 8% higher under-five mortality rate, even after adjusting for distance to the nearest health centre. Internal consistency between the items ranged from moderate to high, with polychoric correlation coefficients ranging from 0.49 (items ‘How long does it take for you to get to this village on rainy season’ and ‘Condition of the roads on non-rainy season’) to 0.94 (items ‘How hard this village is to reach’ and ‘How difficult it is for the residents to seek medical care’). Inter-rater reliability for all-item composite score and two-item composite score, was moderate to strong, with the average ICCs being 0.74 and 0.69, respectively). The composite scores showed expected associations with objective accessibility indicators, with Pearson correlation coefficients between all-item composite score and distance to the nearest health facility, average monthly rainfall, and travel time by car being, 0.36 (*P* < 0.001), 0.22 (*P* < 0.001), and 0.35 (*P* < 0.001), respectively. Pearson correlation coefficients between two-item composite score and distance to the nearest health facility, average monthly rainfall, and travel time by car were 0.34 (*P* < 0.001), 0.22 (*P* < 0.001), and 0.32 (*P* < 0.001), respectively.

**Conclusions:**

Our findings indicate acceptable feasibility and reliability, with moderate convergent and predictive validity. This simple survey can serve as a useful tool for evaluating hard-to-reach areas and guiding resource allocation in public health interventions, including mass drug administration campaigns.

**Registration:**

clinicaltrials.gov: NCT04424511 (parent trial)

Access to healthcare remains a key determinant of child survival and well-being, especially in low- and middle-income countries (LMICs). In this context, greater distance from households to health facilities is known to be associated with higher under-five mortality, lower immunisation coverage, and delayed care-seeking for acute illnesses [1–3]. Recent findings indicate that geographical accessibility can also modify the impact of public health interventions. For instance, the effect of azithromycin mass drug administration on child mortality has been stronger in more remote areas [4]. Bed net ownership was also characterised by vast geographic disparities, highlighting the negative correlation between distance to primary national roads and distance to the nearest health facility [5].

However, distance alone cannot fully explain the complexity of healthcare accessibility. Environmental conditions, such as seasonal flooding, limited transportation, and poor infrastructure, together with social and economic constraints, may critically impede access even for communities relatively close to health facilities [6–9]. Communities with limited access to healthcare can also be more prone to having a higher disease burden, as they face overlapping issues related to poor socio-economic and infrastructural conditions [10–13]. The concept of ‘hard-to-reach’ populations has, therefore, gained prominence, yet remains inconsistently defined and measured [6]. Existing categorisations often rely on spatial data or modelled estimates of remoteness, which are sometimes incomplete or outdated, and may not reflect local realities [14–16]. The *Stratégie Avancée* programme in Mali, for example, classifies villages by distance to health facilities, yet this approach rarely captures the broader contextual factors that make service delivery genuinely difficult [17].

To address these limitations, we developed a simple, locally informed tool to assess geographical hardness-to-reach. Unlike purely distance-based or computational measures, this tool draws on local perceptions of accessibility, allowing for a more context-specific understanding of how geography and infrastructure affect service delivery. Conceptually, ‘hardness-to-reach’ affects access to healthcare and service availability, which in turn affect health outcomes, such as mortality. Remoteness and infrastructure describe structural characteristics of a location, while accessibility reflects the ease of reaching services, and deprivation captures underlying socio-economic disadvantage. Hardness-to-reach thus refers to the combined and context-dependent manifestation of these factors, as experienced in practice. While public health has traditionally focused on tangible outcomes such as mortality, there is growing recognition of the value of subjective measures, such as quality of life and pain [18], as complements to objective indicators [19]. In this context, we sought to propose subjective measurement of hardness-to-reach as a meaningful and informative construct for public health. By quantifying perceived hardness-to-reach, the tool complements existing spatial methods and provides a standardised measure that can inform research and implementation planning. This approach may improve the identification of areas where additional logistical support is needed and contribute to more equitable health interventions in LMICs.

## METHODS

We developed the hardness-to-reach tool (Table S1 in the **Online Supplementary Document**) within this sub-study of Large-scale Assessment of the Key health-promoting Activities of two New Mass Drug Administration regimens with Azithromycin (LAKANA) trial, a multi-year study covering 1,151 villages in Mali [20], as a simple questionnaire to be implemented by frontline data collectors (DCs) in capturing key determinants of hardness-to-reach. The 14 questionnaire items were generated by consulting two field team leaders on the reoccurring difficulties experienced during the trial, such as that accessibility varied by season, travel time and transportation posed challenges, and DCs encountered additional unexpected barriers when travelling to the villages during mass drug administration visits. Items were grouped into four thematic domains: methods of transportation, topographical difficulty, travel time, and overall subjective judgment. The lead investigator drafted the initial tool in English and refined it with a native French-speaking investigator. The draft was then reviewed by field leaders and pilot-tested with five DCs to ensure functionality, clarity in French, and consistency with prior knowledge of the locations.

Survey items are answered using either a dichotomous (yes/no) format or a five-point Likert scale, except for one item that requires specifying the mode of transportation used to reach the village when it was not accessed by car. The five-point scale items were originally coded from 1 (difficult) to 5 (easy) and subsequently reverse-coded in the analysis so that higher scores reflected greater ‘hard-to-reach’ status. Data were captured electronically using the CommCare system (Dimagi, Cambridge, Massachusetts, USA).

### Study population and sample size considerations

We aimed to create a measure that represents a subjective assessment of geographical accessibility of all 1,151 villages included in the trial. We carried out survey rounds out at the end of 2024, coinciding with the conclusion of the trial. The survey was self-administered using data collection tablets given to respondents by the supervisors of field activities. The participants were limited to completing the survey for up to 20 villages at a time, with the option to restart the electronic questionnaire without losing previous evaluations.

To ensure geographic coverage across all 11 districts in which LAKANA operated (Selingue, Sefeto, Sagabari, Koulikoro, Kati, Kita, Kéniéba, Ouéléssébougou, Kangaba, Kalabancoro, and Fana), 25 DCs with experience in one or more of the districts were invited, and all agreed to participate. A brief information sheet for informed consent was provided for the DCs before they were given the questionnaire. However, to preserve confidentiality, the names or positions of the DCs were not recorded in the electronic system and informed consent was obtained verbally. The DCs were not assigned to specific villages to assess, but were rather allowed to select the villages themselves. This helped ground the assessments in direct field experience and, alongside administration through multiple rounds, minimised the respondent burden. After evaluating the villages of their choosing, DCs were asked to assess how laborious the survey process felt.

### Sample size calculation

Assuming concurrent validity in terms of Pearson correlation coefficient (*r*) between the tool score and validity criteria variables was approximately 0.5, rating at least 220 villages would offer a precision level (width of 95% confidence interval (CI)) of ±0.1 for the estimates. This sample size also offered precision level of ±0.05 for inter-rater reliability estimate, *i.e.* the intraclass correlation coefficient (ICC), assuming it to be approximately 0.8.

### Descriptive statistics

We calculated the mean and standard deviation (SD) values for each item and composite scores and evaluated the potential floor and ceiling effects, *i.e.* do survey responses on items accumulate on the highest or lowest value.

### Feasibility

To assess respondent burden, we captured the completion time per village from the digital survey application and asked DCs to rate the burden by choosing one of three options: ‘The questionnaire was laborious’, ‘The questionnaire was moderate’, and ‘The questionnaire was light’.

### Predictive and convergent validity

We used the under-five mortality from LAKANA trial as the outcome for assessing the predictive validity of the tool, as it reflects the cumulative effects of a complex system of health-related factors, and as it is a policy-relevant endpoint that directly aligns with the end goal of saving lives. While individual factors such as treatment seeking behaviour or facility attendance may provide additional information, they were not available for the study area. In LAKANA, DCs visited villages in eight three-month cycles and recorded the vital status of all household members at each visit. For this analysis, we aggregated the sums of deaths and person-time of children under the age of five years per village.

We calculated two composite scores: one including all survey items and a reduced score using only two items – the estimate of village residents’ access to healthcare and the overall estimate of hardness-to-reach. We selected those two items because they were generic and applicable irrespective of settings and users. To test if this minimal item set could approximate the full set, we inspected the Pearson’s *r* between the two-item composite score and all-items composite score, as well as between the two-item composite score and the sum of the remaining items.

We selected objective accessibility indicators for evaluating convergent validity based on existing literature [5,6] and observations from the LAKANA trial, such as heavy rains, deteriorated road or terrain conditions, or long travel distances. The selected indicators were the great circle, or ‘as-the-crow-flies’; distance from the nearest health facility in kilometres; national outreach programme status (*Stratégie Avancée*, standard/advanced); monthly rainfall during the LAKANA trial time frame (2020–2024) in mean mm in month; and travel time to the nearest health facility by car in minutes. We calculated the as-the-crow-flies distances by using aggregated GPS-coordinates of households participating in LAKANA trial and the Haversine method [21] to estimate the distance between a village and the closest health centre. We retrieved rainfall information *via* Open-Meteo [22] and travel times by car *via* OpenStreetMap [23].

We used Poisson mixed-effects models with random intercepts for DCs to assess the association between under-five mortality and both the items and the composite scores, using village-level person-years as an offset variable. To assess the predictive value of the survey responses independent of geographic proximity, additional analyses adjusted for distance to the nearest health facility. We used linear mixed effects regression models with the objective accessibility indicators as dependent variables and the survey responses as independent variables with random intercepts for DCs to evaluate convergent validity. Variables in the model were standardised to have zero-mean and SD of one such that the coefficients have an interpretation of Pearson’s *r*. To evaluate known-group validity, we compared the composite scores between the outreach strategy status using boxplots. We also established a mixed effects model with random intercept for DCs and a logistic link function to evaluate how one-unit change in the composite score relates to the odds of being in the advanced group.

### Internal consistency and inter-rater reliability

We used polychoric correlation to assess relations between survey items, separately for villages that are reachable by vehicle or not, as some items are conditional on that. For inter-rater reliability, we considered only villages assessed by more than one DC, as we wanted to evaluate it distinctly from the performance of the measurement scale in practice. We computed ICCs for each individual Likert scale item on the survey tool, as well as for the composite scores. Because multiple DCs could assess the same village, and because not all 25 DCs evaluated every village, our study had an unbalanced, incomplete observational design for an inter-rater reliability study. Furthermore, we predicted we would have two systematic sources of variance: villages and DCs. Therefore, we used two metrics to evaluate inter-rater reliability: individual ICC and average ICC, calculated from a two-way random-effects model [24–26]. The individual ICC has the interpretation of a single, ‘typical’ DC providing the ratings whereas the average ICC has the interpretation of taking the average rating of multiple ratings on a single village. A commonly used cut-off for acceptable inter-rater reliability ranges between 0.40 and 0.50 [27,28]. Here, we use 0.50 as a cut-off. Technical details of the inter-rater reliability estimation models are described elsewhere (**Online Supplementary Document**).

We carried out all statistical analyses in *R*, version 4.3.2 (R Foundation for Statistical Computing, Vienna, Austria). We report our findings per the STROBE reporting guidelines [29].

## RESULTS

### Feasibility

There were 1,697 assessments covering 1,142 villages, of which 388 were rated by at least two DCs. The mean number of assessments per village was 1.49; villages with only one assessment and those with multiple assessments were spatially evenly distributed (Figure S1 and Table S2 in the **Online Supplementary Document**). A single DC assessed approximately 68 villages, spreading through 4–6 survey rounds of about 15 village assessments. Completing the all-item survey form for a single village took approximately 3 minutes (range = 1.5–7). When DCs assessed fewer than 10 villages in a round, respondent burden was rated as light, moderate, and laborious in 29 (60%), 9 (19%), and 10 (21%) response rounds, respectively. In rounds with 10 or more villages, respondent burden was rated as light, moderate, and laborious in 40 (43%), 14 (15%), and 39 (42%) response rounds, respectively.

### Descriptive statistics

The mean values of the all-items and two-item composite scores were 22.4 (SD = 6.4) and 6.2 (SD = 1.9), respectively (**Table 1**). In the thematic domain of subjective judgement, the items of access to healthcare, overall estimate, and equipment transportation difficulty in rainy and non-rainy seasons, had mean values of 3.1 (SD = 1.0), 3.1 (SD = 1.0), 3.4 (SD = 1.1), and 2.8 (SD = 1.0), respectively. Regarding clustering of values on either on the lowest or the highest, there was little floor or ceiling effect, except that 17.4% of the ratings on the item on equipment transportation difficulty in rainy season were at the ceiling.

**Table 1:** Descriptive statistics of the survey scores on 1,697 village assessments

	Thematic domain	Possible range	x̄ (SD)	% at max	% at min
**Composite score, all items**	Composite score	8–38*	22.4 (6.4)	2.0	2.7
**Composite score, two items**	Composite score	2–10†	6.2 (1.9)	2.0	2.7
Estimate of village residents’ access to healthcare	Subjective judgement	1–5	3.1 (1.0)	8.5	5.1
Overall estimate of hardness to reach	Subjective judgement	1–5	3.1 (1.0)	6.7	4.8
How difficult is it to transport equipment to the village in the rainy season?	Subjective judgement	1–5	3.4 (1.1)	17.4	3.3
How difficult is it to transport equipment to the village in the non-rainy season?	Subjective judgement	1–5	2.8 (1.0)	2.9	10.0
Difficulty of the terrain on non-rainy season	Topographical difficulty	1–5	4.1 (0.8)	32.7	1.8
Difficulty of the terrain on rainy season	Topographical difficulty	1–5	4.6 (0.5)	56.3	0.0
Condition of the roads on rainy season	Topographical difficulty	1–5	3.4 (1.1)	16.7	3.4
Condition of the roads in the non-rainy season	Topographical difficulty	1–5	2.7 (0.9)	1.3	10.5
Estimate of travel time to the village on rainy season	Travel time	1–5	1.7 (0.7)	0.2	44.3
Estimate of travel time to the village on non-rainy season	Travel time	1–5	1.3 (0.6)	0.2	69.8
Can the village be reached by large, motorised vehicle?	Method of transportation	No = 1, yes = 0	96.8 (17.6)‡	3.2	96.8
Are there any public transportation possibilities for the residents?	Method of transportation	No = 1, yes = 0	79.1 (40.7)‡	79.1	20.9

SD – standard deviation, x̄ – mean

*8 = Very easy to reach, 38 = very difficult to reach.

†2 = very easy to reach, 10 = very difficult to reach.

‡Expressed in %.

Approximately 3.2% of the responses indicated that the village was not reachable by car (alternative reported methods of transportation for reaching the village are listed in Table S3 in the **Online Supplementary Document**), while 79.1% indicated that there are no public transportation possibilities for the residents. Within the thematic domain of topographical difficulty, the mean score of was 3.4 (SD = 1.1) for the item ‘Condition of the roads on rainy season’ and 2.7 (SD = 0.9) for the item ‘Condition of the roads on non-rainy season’. There was ceiling effect in the items, whereby 16.7% and 13.0% of the ratings per each item, respectively, were at the ceiling.

The mean estimated travel time between the village and health centre was 2–3 (SD = 1) hours during the rainy season and 1–2 (SD = 1) hours during non-rainy season. There was floor effect regarding the travel time, with 44.3% and 69.8%, respectively, being at the floor, which indicated travel time being less than one hour.

### Internal consistency

Polychoric correlation coefficients among the items were ≥0.49. The highest correlation (0.94) was observed between ‘access to healthcare’ and ‘overall estimate of hardness-to-reach’, while the lowest (0.49) was between ‘travel time estimate on rainy season’ and ‘road condition estimate during non-rainy season’ (**Figure 1**; Figure S2 in the **Online Supplementary Document**). The two-item composite score showed a correlation of 0.94 with the all-item composite score and 0.89 with the sum of remaining items.

**Figure 1:**
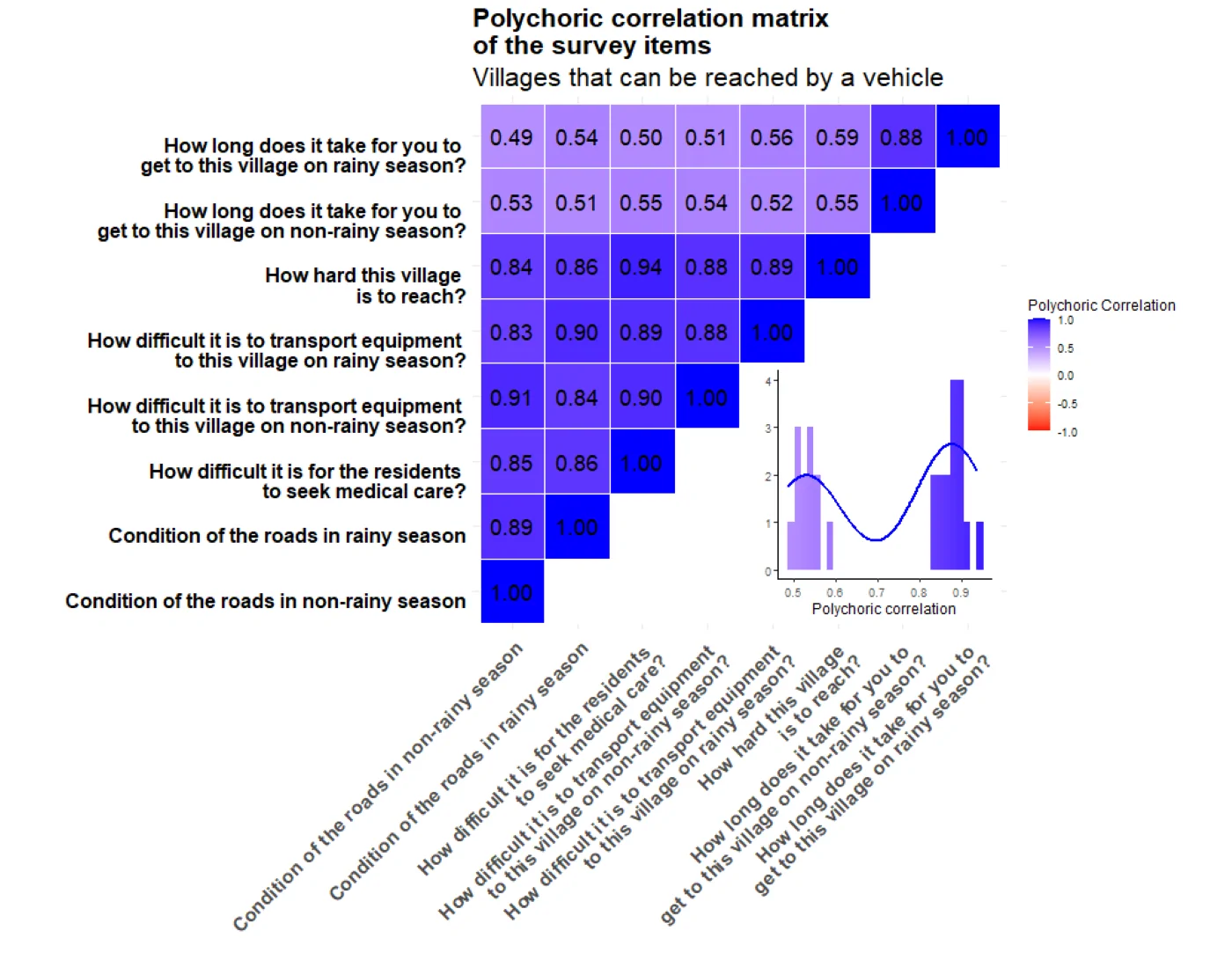
Polychoric correlation matrix of the five-point survey items for villages that can be reached by a vehicle.

### Predictive, known group, and convergent validity

The mixed-effects model yielded an unadjusted risk ratio (RR) of 1.17 (95% CI = 1.12–1.21, *P* < 0.001) for the association between the all-items composite score and under-five mortality (**Table 2**). After adjustment for distance to nearest health facility, the adjusted RR was 1.08 (95% CI = 1.03–1.13, *P* < 0.001). For the two-item composite score, the unadjusted RR was 1.16 (95% CI = 1.11–1.20, *P* = 0.001) and the distance-adjusted RR was 1.07 (95% CI = 1.03–1.12, *P* = 0.001).

**Table 2:** Association between under-five mortality rate of villages and two versions of survey composite score (higher score means harder to reach) and distance to nearest health facility

				

Model*	Independent variables	Unadjusted RR per SD (95% CI)	Adjusted RR per SD (95% CI)	Adjusted *P*-value
Model 1	All-item composite score	1.17 (1.12–1.21)	1.08 (1.03–1.13)	<0.001
	Distance to health facility†		1.19 (1.14–1.25)	<0.001
Model 2	Two-item composite score	1.16 (1.11–1.20)	1.07 (1.03–1.12)	0.001
	Distance to health facility†		1.20 (1.15–1.25)	<0.001

aRR – adjusted risk ratio, CU – confidence interval, RR – risk ratio, SD – standard deviation

*Models 1 and 2 indicate either all-item or two-item variable being the main independent variable.

†SD = 4.7.

The ‘advanced’ category in outreach strategy had a mean all-items composite score of 23.7, while the ‘standard’ category had a mean composite score of 19.0 (**Figure 2**). Furthermore, an increase in the composite score was associated with an increase in the odds of belonging to the advanced group; the odds ratio per SD increase in the composite score was 2.32 (95% CI = 2.03–2.65, *P* < 0.001). The means of two-item composite score in the advanced and standard categories were 6.5 and 5.3, respectively, and the odds ratio for one SD increase in the two-item score was 2.05 (95% CI = 1.81–2.35, *P* < 0.001).

**Figure 2:**
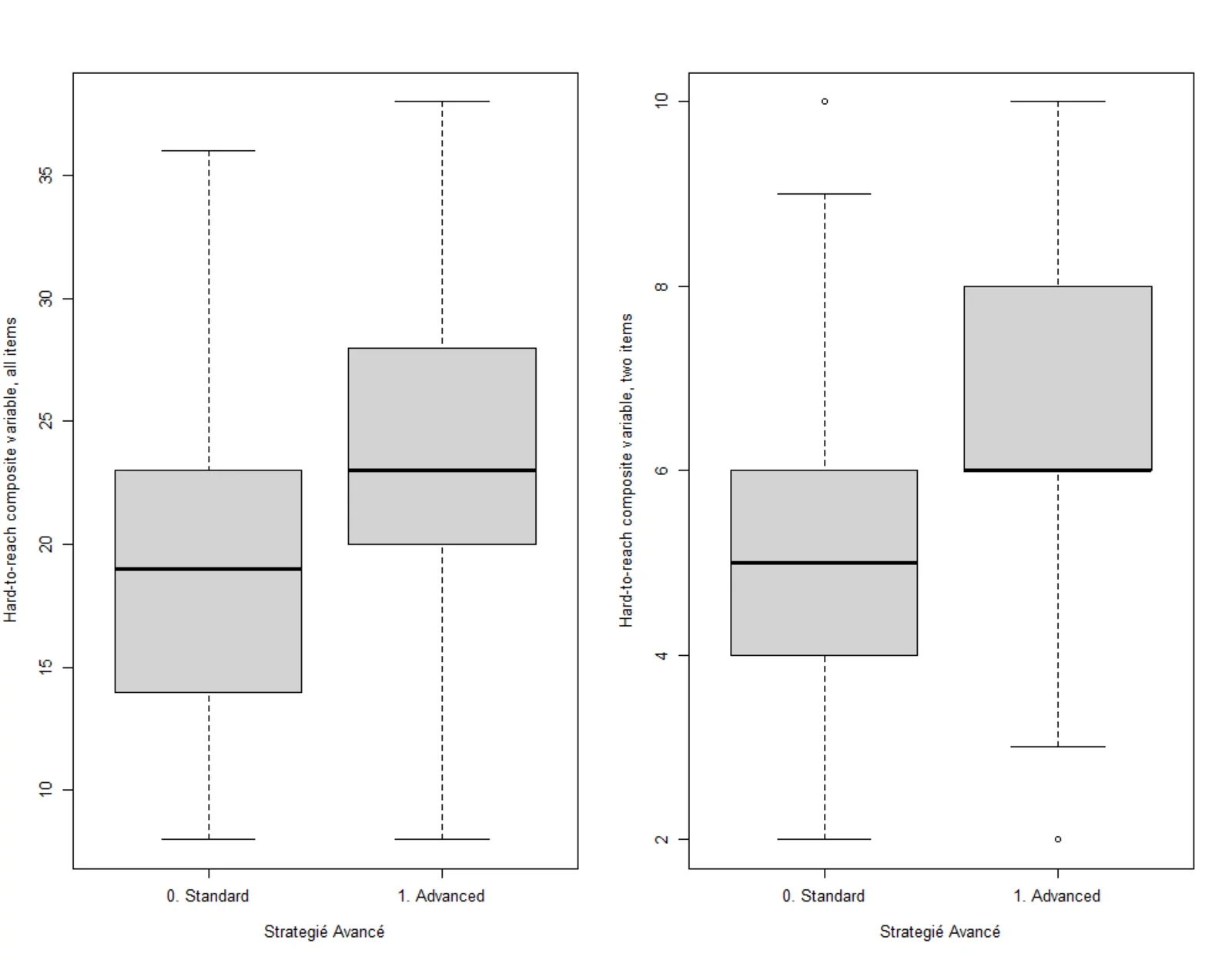
Boxplot visualisation of the key variable values in ‘standard’ and ‘advanced’ *Stratégie Avancée *groups.

Regarding convergent validity, the all-item composite score was significantly correlated with the as-crow-flies distance to nearest health facility (Pearson’s *r* = 0.36; 95% CI = 0.31–0.40, *P* < 0.001), average monthly rainfall (Pearson’s *r* = 0.22; 95% CI = 0.16–0.26, *P* < 0.001), and travel time to the nearest health facility by a vehicle (Pearson’s *r* = 0.35, 95% CI = 0.30–0.39). The two-item composite score was significantly correlated with the as-the-crow-flies distance to nearest health facility (Pearson’s *r* = 0.34; 95% CI = 0.30–0.39, *P* < 0.001), average monthly rainfall (Pearson’s *r* = 0.22; 95% CI = 0.17–0.27, *P* < 0.001), and travel time to the nearest health facility by a vehicle (Pearson’s *r* = 0.32; 95% CI = 0.27–0.37) (Table S4 in the **Online Supplementary Document**).

### Inter-rater reliability

The individual ICC of all-item composite score and two-item composite score were 0.57 (95% CI = 0.51–0.63) and 0.51 (95% CI = 0.44–0.57), respectively (**Table 3**). Most of the other items in the survey gave individual ICC values between 0.4 and 0.5.

**Table 3:** ICC on each five-point scale item and the composite score

	Individual ICC (95% CI)	Average ICC (95% CI)
**Composite variable, all items**	0.57 (0.51–0.63)	0.74 (0.68–0.78)
**Composite variable, two items**	0.51 (0.44–0.57)	0.69 (0.62–0.74)
Estimate of village residents’ access to healthcare	0.49 (0.42–0.55)	0.66 (0.60–0.72)
Overall estimate of hardness to reach	0.49 (0.41–0.55)	0.67 (0.60–0.72)
How difficult is it to transport equipment to the village in the rainy season?	0.48 (0.40–0.54)	0.65 (0.58–0.71)
How difficult is it to transport equipment to the village in the non-rainy season?	0.46 (0.39–0.53)	0.65 (0.58–0.70)
Difficulty of the terrain on rainy season	0.64 (0.05–0.99)	0.68 (0.06–0.99)
Difficulty of the terrain on non-rainy season	0 (-0.64–0.63)	0 (-0.80–0.64)
Condition of the roads on rainy season	0.41 (0.33–0.47)	0.58 (0.50–0.65)
Condition of the roads in the non-rainy season	0.41 (0.33–0.48)	0.58 (0.49–0.65)
Estimate of travel time to the village on rainy season	0.32 (0.25–0.39)	0.50 (0.41–0.58)
Estimate of travel time to the village on non-rainy season	0.23 (0.15–0.30)	0.39 (0.27–0.48)

CI – confidence interval, ICC – intra-class coefficient

The harmonic mean number of DCs per village was 2.30. The all-item and two-item composite scores had average ICCs of 0.74 and 0.69, respectively. Similarly, most other items had average ICCs about 0.2 larger than their individual ICC. The difference indicates the improved performance of the measure by using 2–3 DCs to rate a village as compared to using only one DC to conduct the assessment.

## DISCUSSION

We developed and evaluated a new tool designed to measure the level of geographical hardness-to-reach of villages and evaluated its feasibility and measurement performance. Each DC who participated in the implementation of the tool had worked in the LAKANA trial in Mali and, although they rated multiple villages, they generally considered the survey to be feasible to implement in terms of respondent burden. Markedly, survey items and their derived composite scores indicated a 4–8% higher under-five mortality per one SD change, even when the model was adjusted for the distance from the nearest health facility, a usual predictor of increased under-five mortality. The tool’s composite scores were associated with national outreach programme, *Stratégie Avancée* [17], classifications in an expected manner. The ‘advanced’ category had higher composite scores than the ‘standard’ category, indicating that the score effectively captures dimensions of accessibility. Convergent validity was supported by statistically significant associations between the composite score and objective indicators, including as-the-crow-flies distance and average monthly rainfall. While we did not pre-specify which level of coefficients would be acceptable, referring to the conventional classifications of 0.1, 0.3 and 0.5 as small, medium, and large [30] would suggest that our results indicate a medium level of evidence for convergent validity.

Subjective ratings and objective indicators may overlap, so the former may better be considered complementary, rather than as a replacement for the latter. Indeed, these findings indicate that the tool may capture meaningful aspects of geographical accessibility not otherwise captured by simple distance metrics, which often overlook barriers such as road conditions, terrain, weather, and transport availability [31,32]. They could offer information relevant to strategic planning and insight for targeting health interventions in remote areas.

The two-item composite score correlated strongly with both the all-item composite score and the sum of remaining items, which indicates that the two-item version of the tool may be a meaningful approximation of the full-length tool. Furthermore, we found that the internal consistency among survey items was high and the individual inter-rater reliability was moderate, with the latter having the potential to be further improved by using multiple raters to assess the same locations, as average ICC values were stronger and highest for the composite scores. However, even individual ICCs for both composite scores had point estimates above 0.50.

Several limitations should be noted. First, and perhaps most importantly, the survey was conducted at the end of the trial period, meaning that most assessments were based on retrospective recall, rather than real-time observation. This introduces the possibility of recall bias [33]: since the DCs were in contact with the village residents, the residents’ reports of health outcomes may have influenced the DC’s evaluations. The retrospective dimension can also produce recall error [34]: since LAKANA was a multiyear trial, the DCs may have experienced inaccuracies for villages that were visited earlier due to the time since the visit. It is also possible that local infrastructure and circumstances have shifted since the visit, making the subjective evaluation outdated. Nevertheless, in villages evaluated by multiple DCs, inter-rater reliability was moderate, with higher ICCs for composite scores, indicating a consistent underlying signal despite individual variability. Recall errors are unlikely to be systematically aligned across DCs and are more plausibly driven by individual differences. Aggregating ratings is therefore expected to reduce such error and improve robustness. Operational factors may also have influenced results, and immediate assessment during visits could improve reliability, which warrants further study.

The item generation process was not comprehensive, as cognitive interviewing was not conducted and only one round of pilot testing was performed. However, the items were designed to be simple and unambiguous, which reduced the likelihood of misinterpretation. The pilot also yielded positive feedback from the five participating DCs. Furthermore, the DCs selected the villages they evaluated to ensure familiarity, which introduces potential selection bias. However, its impact is likely limited, as each DC assessed a large number of villages and overall coverage reached 99%. While some bias cannot be excluded, it is unlikely to have materially influenced the findings.

The terrain difficulty items showed low variability, leading to near-singular mixed-effects models with variance components close to zero. Consequently, the non-rainy season item yielded an ICC close to zero, while the rainy season item showed wide 95% CIs. Limited variability was also indicated by observed ceiling effect. Despite slightly less ceiling effect in the non-rainy item, within-village variability remained low, contributing to unstable ICC estimates, including negative 95% CI bounds.

Individual items rated by a single respondent yielded ICCs of approximately only 0.5. Given that the ICC can be interpreted as a sample size issue due to measurement imprecision [35], the use of the individual item rated by a single respondent requires doubling the sample size to achieve the same power or precision. In contrast, the use of composite scores and multiple raters increased ICC values to approximately 0.7. These findings should be considered when selecting items and designing data collection strategies, particularly with regard to the use of composite measures and the number of raters.

Although internal consistency was high, polychoric correlation exceeding 0.9 between ‘overall estimate of hardness-to-reach’ and ‘access to healthcare’ suggests potential redundancy. However, the items capture distinct dimensions, as a village may have good health access but still be considered hard-to-reach due to remoteness. Finally, while the *Stratégie Avancée* is an informative component for known-group validity analysis, it overlaps conceptually with the tool, potentially inflating group differences. These findings should, therefore, be interpreted as supportive, rather than definitive, and thus warrant future validation using more independent external criteria.

The number of DCs involved in the survey was relatively small. Despite this limitation, the study benefited from the geographic diversity covered by the DCs, who collectively provided assessments across a wide range of environments. To address the unbalanced and incomplete design, we applied current methodological recommendations for estimating ICCs [24].

A secondary analysis of the MORDOR trial found that each additional kilometre of distance from a health facility was associated with an approximately 5% increase in the incidence rate ratio of under-five mortality, with 95% CIs ranging from 3% to 7% [4]. Our results align with this finding when they are transformed to kilometre scale from SD scale. This is particularly relevant when considered in the context of scale up activities of azithromycin mass drug administration programming from Resiliency through Azithromycin for Child Survival network [36–39] and this tool could support ongoing efforts to identify and prioritise hard-to-reach populations. Moreover, as the importance of engagement of community stakeholders is widely recognised [40], and as the tool relies upon the subjective assessment of local individuals, the usage of this tool could bring into the implementation an aspect of engagement of the raters. For example, health workers, school teachers, outreach vaccine workers, and volunteers would act as co-investigators, rather than simply data collectors. In this context, a key contribution of this work is to propose and validate the concept that subjective measurement of hardness-to-reach can contribute information relevant to public health. It also provides relevant information for future researchers to make informed choices who would want to use either the full tool, composite scores, or single items. However, more validation research is needed to establish its usability across countries, personnel backgrounds, and cultural contexts.

## CONCLUSIONS

This study has demonstrated the validity and reliability of the proposed tool for subjective assessment of geographical hardness-to-reach of communities and its ability to differentiate locations with varying mortality levels. A two-item composite score can improve feasibility and reduce respondent burden without much loss in validity and reliability, as compared to using the version of composite score based on 14 items. Some of the 14 items were specific to the LAKANA trial context such as those related to transportation of equipment. In contrast, the two-item composite score relies on generic items that are broadly applicable across settings. Therefore, we see higher applicability of the two-item versions, with the caveat that further evidence is needed to establish performance across contexts. Future validation in other low-income settings and possibly other types of raters will be valuable for further refinement.

## Additional material

Online Supplementary Document

## Data Availability

**Data availability:** The data are available *via* Zenodo (https://doi.org/10.5281/zenodo.18312097) upon reasonable request to the LAKANA Trial Principal Investigators at lakana.psg@lists.tuni.fi. It is aggregated at a pseudonymised cluster level and contains no information traceable to individuals. This Zenodo project includes the LAKANA hard-to-reach data set.csv (aggregated cluster-level data from LAKANA and hard-to-reach tool evaluations) and the LAKANA hard-to-reach data dictionary.xlsx (variable descriptions). The data are available under the terms of the Creative Commons Attribution 4.0 International license. The underlying analytical code for the hard-to-reach study is available at GitHub (https://github.com/LAKANA-Trial-team/Hard-to-reach/tree/v3.0.0) and archived on Zenodo (https://doi.org/10.5281/zenodo.18325366). The code is distributed under an OSI-approved open-source license.
