# Supplementary material for: Development and evaluation of a subjective measurement of geographical hardness-to-reach: a survey of data collectors in a large-scale cluster randomised trial of azithromycin mass drug administration
Source: J Glob Health. 2026 Nov 6;16:04322. doi: 10.7189/jogh.16.04322 (PMC13741166; doi:10.7189/jogh.16.04322)
Supplement: Supplementary file 1 [file jogh-16-04322-s001.pdf]

Supplement to: Luoma J, Haidara FC, Samake O, Adubra L, Abdou M, Alber D, Ashorn U, Cloutman-Green E, Diallo M, Juma J, Ducker C, Fan Y, Gruffud G, Hallamaa L, Ihamuotila R, Martell O, Okello C, Haapaniemi T, Vehmasto T, Murugesan A, Traore CAT, Ylikruuvi K, Klein N, Ashorn P, Sow S, Cheung YB. Development and evaluation of a subjective measurement of geographical hardness-to-reach: a survey of data collectors in a large-scale cluster randomised trial of azithromycin mass drug administration. J Glob Health. 2026;16:04322.

Table of Contents

Supplementary Table 1 2
Supplementary Table 2 2
Supplementary Table 3 3
Supplementary Table 4 4
Statistical models of ICC 5

Supplementary Table 1

Item		Theme	Values
1.	Can the village be reached by large, motorized vehicle?	Method of transportation	Yes/No
2.	If the village can't be reached by car, how can you reach the village during non-rainy season?	Method of transportation	By Boat;<br>By Scooter;<br>By Bicycle;<br>By Foot
3.	If the village can't be reached by car, how can you reach the village during rainy season?	Method of transportation	By Boat;<br>By Scooter;<br>By Bicycle;<br>By Foot
4.	Are there any public transportation possibilities for the residents?	Method of transportation	Yes/No
5.	Difficulty of the terrain on non-rainy season	Topographical difficulty	5-point scale
6.	Difficulty of the terrain on rainy season	Topographical difficulty	5-point scale
7.	Condition of the roads on rainy season	Topographical difficulty	5-point scale
8.	Condition of the roads in the non-rainy season	Topographical difficulty	5-point scale
9.	Estimate of travel time to the village on rainy season	Travel time	5-point scale
10.	Estimate of travel time to the village on non-rainy season	Travel time	5-point scale
11.	How difficult is it to transport equipment to the village in the rainy season?	Subjective judgement	5-point scale
12.	How difficult is it to transport equipment to the village in the non-rainy season?	Subjective judgement	5-point scale
13.	Overall estimate of hardness to reach	Subjective judgement	5-point scale
14.	Estimate of village residents' access to health care	Subjective judgement	

Supplementary Table 2

Descriptive numbers on villages, fieldworkers, and assessments

Metric	
Number of villages	1142
Number of raters	25
Number of Assessments	1697
Average number of village assessments per rater	68
Average number of villages assessed in a single electric form	15
Average number of Assessments per village	1.49
Average time (minutes) of assessing a single village in a survey	3

Supplementary Table 3

Number of reported answers of methods of reaching the village when the village is unreachable by car.

Method of access, non-Rainy season		
	Bicycle	3
	Boat	2
	Foot	11
	Scooter	52
Method of access, rainy season		
	Bicycle	2
	Boat	2
	Foot	12
	Scooter	51

Supplementary Table 4

Associations between the composite scores and the objective indicators.

Composite score	Objective indicator	Pearson's r (95% CI)	P-value
All-item composite score	Distance to the nearest health facility	0.36 (0.31 to 0.40)	<0.001
Two-item composite score		0.34 (0.30 to 0.39)	<0.001
All-item composite score	Average monthly rainfall	0.22 (0.18 to 0.27)	<0.001
Two-item composite score		0.22 (0.17 to 0.27)	<0.001
All-item composite score	Travel time by car	0.35 (0.30 to 0.39)	<0.001
Two-item composite score		0.32 (0.27 to 0.37)	<0.001

Statistical models of ICC

There are many coefficients available for measuring inter-rater reliability, but as highlighted by Ten Hove et al. [1], the different indices have substantially different interpretation regarding the quality of inter-rater reliability. For our purposes, we decided to use ICC as the index as it has intuitive connection to the classical test theory (CTT) framework [2,3].

2,3

Within CTT, reliability is defined as the ratio of true score variance σ_T^2 and the observed score variance σ_X^2. Since the observed score can be expressed as the sum of the true score and error, the reliability under CTT framework can be expressed as

$$\rho_{XT} = \frac{\sigma_T^2}{\sigma_X^2} = \frac{\sigma_T^2}{\sigma_T^2 + \sigma_E^2}$$

The formulation is the same as the "classical" ICC formula presented by Fisher already in 1925 [4], and later referred to as ICC(1,1) model by Shrout & Fleiss [5].

In our study, the "classical" one-way model ICC wasn't appropriate as we have two systematic sources of variance: subjects and raters. To this end, we chose to use two-way models to calculate both individual and average ICC values for the items, as their respective values could provide insight on both potential use cases. That is, a single assessor (e.g., a data collector) independently evaluating village status, and a team-based assessment model, such as in the LAKANA trial, where multiple field workers assess a village and the average of their ratings determines its classification as hard-to-reach [3,6]. Leaning on the existing literature [3,5,6], we used two models to evaluate consistency among assessors:

individual $ICC(Q,1) = \frac{\sigma_s^2}{\sigma_s^2 + Q\sigma_r^2 + \sigma_{sr}^2}$

and

average $ICC\left(Q,\hat{k}\right) = \frac{\sigma_s^2}{\sigma_s^2 + Q\sigma_r^2 + \frac{\sigma_{sr}^2}{\hat{k}}}$,

where σ_s^2 is the variance between the villages (or "subjects"), σ_r^2 is the variance between the raters, σ_{sr}^2 is the variance of the village-rater interaction, Q is the proportion of nonoverlap across raters, and $\hat{k}$ is the harmonic-mean number of raters. The harmonic-mean accommodates the incomplete design, where every village is not assessed by the same field workers; harmonic-mean number of raters expresses how many field workers on average evaluated an individual village. Q reflects the proportion of non-overlapping raters which impacts the rater variance. We used here the same notation as Ten Hove et al. [3], and we refer the reader to their work for the exact details. A practical documentation of their work has been documented in R language[7].

REFERENCES

1. Ten Hove D, Jorgensen TD, Van Der Ark LA. On the Usefulness of Interrater Reliability Coefficients. In: Wiberg M, Culpepper S, Janssen R, González J, Molenaar D, editors. Quantitative Psychology [Internet]. Cham: Springer International Publishing; 2018 [cited 2025 June 24]. p. 67–75. (Springer Proceedings in Mathematics & Statistics; vol. 233). Available from: http://link.springer.com/10.1007/978-3-319-77249-3_6

2. Lord FM, Novick MR, Birnbaum A. Statistical theories of mental test scores. Vol. 1968. Addison-Wesley;

3. Ten Hove D, Jorgensen TD, Van Der Ark LA. Updated guidelines on selecting an intraclass correlation coefficient for interrater reliability, with applications to incomplete observational designs. Psychological Methods. 2024 Oct;29(5):967–79.

4. Fisher RA. Statistical Methods for Research Workers. In: Kotz S, Johnson NL, editors. Breakthroughs in Statistics [Internet]. New York, NY: Springer New York; 1992 [cited 2025 June 24]. p. 66–70. (Springer Series in Statistics). Available from: http://link.springer.com/10.1007/978-1-4612-4380-9_6

5. Shrout PE, Fleiss JL. Intraclass correlations: Uses in assessing rater reliability. Psychological Bulletin. 1979;86(2):420–8.

6. McGraw KO, Wong SP. Forming inferences about some intraclass correlation coefficients. Psychological Methods. 1996 Mar;1(1):30–46.

7. ten Hove D, Jorgensen TD, van der Ark LA. Updated Guidelines on Selecting an ICC for IRR. 2021 [cited 2026 Jan 21]; Available from: https://osf.io/8j26u/
